# Comparative Analysis of Antioxidant System and Salt-Stress Tolerance in Two Hibiscus Cultivars Exposed to NaCl Toxicity

**DOI:** 10.3390/plants12071525

**Published:** 2023-03-31

**Authors:** Wenjing Lu, Ye Zhao, Jinying Liu, Bowen Zhou, Guoqing Wei, Ruiqiang Ni, Shuyong Zhang, Jing Guo

**Affiliations:** College of Forestry, Shandong Agricultural University, Tai’an 271018, China

**Keywords:** *Hibiscus syriacus*, salt stress, ionic imbalance, oxidative stress, organic osmolytes

## Abstract

Hibiscus (*Hibiscus syriacus* L.) is known as a horticultural plant of great ornamental and medicinal value. However, the effect of NaCl stress on hibiscus seedlings is unclear. Little is known about *H. syriacus* ‘Duede Brabaul’ (DB) and *H. syriacus* ‘Blueberry Smoothie’ (BS). Here, the effects of solutions with different concentrations of NaCl on the organic osmolytes, ion accumulation, and antioxidant enzyme activity of hibiscus seedling leaves were determined. The results showed that the Na^+^/K^+^ ratio was imbalanced with increasing NaCl concentration, especially in BS (range 34% to 121%), which was more sensitive than DB (range 32% to 187%) under NaCl concentrations of 50 to 200 mM. To cope with the osmotic stress, the content of organic osmolytes increased significantly. Additionally, NaCl stress caused a large increase in O_2_·^−^ and H_2_O_2_, and other reactive oxygen species (ROS), and antioxidant enzyme activity was significantly increased to remove excess ROS. The expression level of genes related to salt tolerance was significantly higher in DB than that in BS under different NaCl concentrations. Taken together, DB possessed a stronger tolerance to salt stress and the results suggest membrane stability, Na^+^/K^+^, H_2_O_2_, catalase and ascorbate peroxidase as salt tolerance biomarkers that can be used for gene transformation and breeding in future hibiscus research.

## 1. Introduction

Soil salinization, one of the major abiotic stress factors, noticeably limits plant growth and development, and reduces productivity [1,2]. More than 800 million hectares of land worldwide are affected by salinization, accounting for more than 6% of the world’s land area [3]. The area of saline-alkali land in China ranks third in the world. The saline-alkali soil in China is mainly distributed in the northwest, northeast, and north China, and there are nearly 2 million hectares of saline soil in the eastern coastal areas [4]. Soil salinity produces osmotic stress in plants and causes the destruction of intracellular dynamic equilibrium and ion distribution [5,6]. In addition, it causes oxidative stress and damages the integrity of the biomembrane system [7]. Furthermore, soil salinity activates signaling cascades that lead to changes in gene expression [8]. In recent years, plant growth responses to salt stress have received increasing attention.

NaCl pressure causes damage to cell membranes, membrane permeability impairment, and aggravation of membrane lipid peroxidation [9]. This abiotic stress also results in an osmotic imbalance in plant cells. To maintain normal life activities, plants need to synthesize proline, soluble sugar, soluble protein, and other organic osmotic substances for osmotic regulation to avoid or mitigate damage [10]. In addition, under salt stress, plants will absorb and accumulate inorganic osmotic regulatory substances, such as Na^+^, K^+^, Ca^2+^, Cl^−^, and other inorganic ions, in cells to regulate osmotic potential [11,12]. However, glycophytes, like hibiscus, do not regularly accumulate Na^+^ and Cl^−^ for osmotic adjustment, as in the case of halophytes [13]. The accumulation of Na^+^ reduces soil osmotic potential and hinders water uptake by roots, resulting in physiological drought in plants [14]. In addition, excessive Na^+^ concentrations hinder the uptake of K^+^, thus disrupting the ion homeostasis of cells and eventually leading to cell death [15].

Under normal conditions, reactive oxygen species (ROS) in plants are in a dynamic equilibrium state with constant production and elimination [16]. When exposed to salt stress, a large amount of ROS will accumulate in plants [17]. Excessive ROS will trigger membrane lipid peroxidation, causing damage to the plant cell membrane system and metabolic process, and oxidative damage to the photosynthetic apparatus [18]. O_2_·^−^ can be converted to H_2_O_2_ and then to OH·, which has a stronger oxidation capacity [19]. Under salt stress, improvement of the scavenging capacity of ROS is one of the important strategies to maintain the normal growth of plants under salt stress [20,21]. Under salt stress, plants usually enhance the activities of antioxidant enzymes and accelerate the scavenging of superoxide free radicals to maintain the balance of active oxygen metabolism and protect the integrity and function of the membrane structure [22,23].

Hibiscus (*Hibiscus syriacus* L.) belongs to the family Malvaceae. Hibiscus is known as a horticultural plant of great ornamental and medicinal value [24]. For example, it has large flowers, variable flower colors and patterns; a three-month flowering period in summer and autumn, and therapeutic effects on certain diseases such as dysentery, cancer, and scabies [25,26,27]. Furthermore, hibiscus has strong drought resistance, cold resistance, and salt tolerance and is an excellent sand-fixing and water- and soil-conservation tree with important ecological value [28,29]. In recent years, scholars have mainly studied the medicinal value of hibiscus [25,26,27]. At present, there are few studies on salt stress in hibiscus, and the biochemical mechanism of hibiscus response to salt stress is still not clear. It is of great significance to study the tolerance mechanism of hibiscus to salt stress for rational planting and popularization in the saline-alkali area of northern China.

We hypothesized that salt-tolerant hibiscus cultivar has the ablility to resist Na^+^ toxicity by maintaining the balance of the antioxidant system and osmotic homeostasis. To test this hypothesis, we investigated the impact of NaCl stress on physiological and biochemical characteristics in two different salt-sensitive hibiscus cultivars grown in North China in this study. The response of lipid peroxidation, organic osmolytes, ion accumulation, and the antioxidant system of two hibiscus cultivars plants with increasing NaCl stress concentration was analyzed to determine the salt tolerance mechanisms of the hibiscus. The resulting information will provide a theoretical basis for the popularization and cultivation of *Hibiscus syriacus* L. in the salinized area of China, thereby increasing its ecological and economic benefits.

## 2. Results

### 2.1. Phenotypes

**Figure 1:**
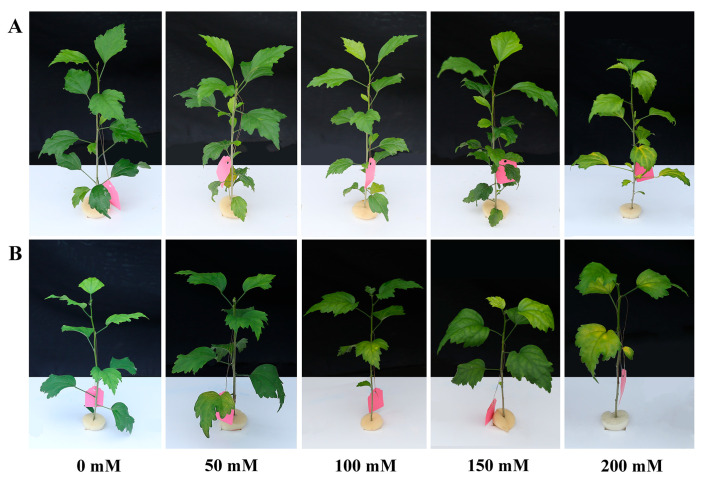
Effect of different NaCl concentrations on the cuttages of two hibiscus cultivars: (**A**) *H. syriacus* ‘Duede Brabaul’ (DB) and (**B**) *H. syriacus* ‘Blueberry Smoothie’ (BS).

With 50 mM NaCl treatment, neither DB nor BS plants exhibited any obvious phenotypic variation (Figure 1). Under the 100 mM NaCl treatment, DB leaves did not change significantly, but chlorotic spots appeared in the middle of the leaves of BS. When treated with 150 mM NaCl, DB plants showed yellowish leaves, but BS leaves showed severe curling and chlorosis. After 200 mM NaCl treatment, DB and BS leaves were severely damaged, and BS leaves fell off. The increase in NaCl stress concentration caused an aggravation of leaf impairment in both DB and BS. However, the degree of phenotypic damage in BS was significantly higher than that in DB under the same NaCl concentration.

### 2.2. Salt Stress Induced Lipid Peroxidation

**Figure 2:**
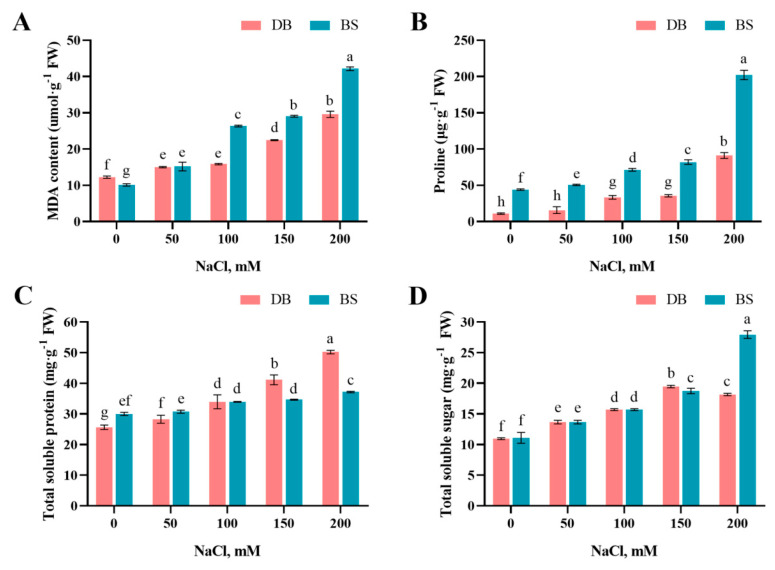
Effect of different NaCl concentrations on (**A**) malondialdehyde (MDA) content, (**B**) proline, (**C**) total soluble sugar, and (**D**) total soluble protein of *H. syriacus* ‘Duede Brabaul’ (DB) and *H. syriacus* ‘Blueberry Smoothie’ (BS). According to Duncan’s test, different lowercase letters (a–g) indicate significant differences (*p* < 0.05) between different treatments.

Lipid peroxidation in the two hibiscus cultivars, which was measured as the malondialdehyde (MDA) concentration, is presented in Figure 2. With increasing NaCl concentration, the MDA content of the two hibiscus cultivar leaves showed an upward tendency (Figure 2A). Except for the 50 mM NaCl treatment, the accumulated MDA content in BS leaves was significantly higher than that in DB leaves under the same concentration treatment.

### 2.3. Salt-Stress-Induced Proline, Soluble Protein, and Soluble Sugar Accumulation

With increasing NaCl concentration, the proline content of the two hibiscus varieties showed an upward trend (Figure 2B). Under the 200 mM NaCl treatment, the accumulated proline content of DB and BS leaves reached a maximum, which increased approximately 8.28 and 4.58 times that of their control, respectively. Although the maximum proline content of BS (202.2 μg·g^−1^) was significantly higher than that of DB (91.1 μg·g^−1^), the increase in proline content of DB was still significantly higher than that of BS. The soluble sugar (TSS) content of DB and BS leaves under NaCl treatment was significantly higher than that of control plants (*p* < 0.05), and it is worth noting that the content of DB was significantly decreased while that of BS was significantly increased to a great extent under 200 mM NaCl treatment compared with 150 mM NaCl treatment (Figure 2C). The soluble protein (TSP) content of DB and BS leaves was also significantly increased under NaCl treatment (*p* < 0.05), while the accumulated soluble protein content of DB leaves was significantly higher than that of BS when high salt stress (150 mm, 200 mm) was applied (Figure 2D).

### 2.4. Salt-Stress-Induced Ion Imbalance

**Figure 3:**
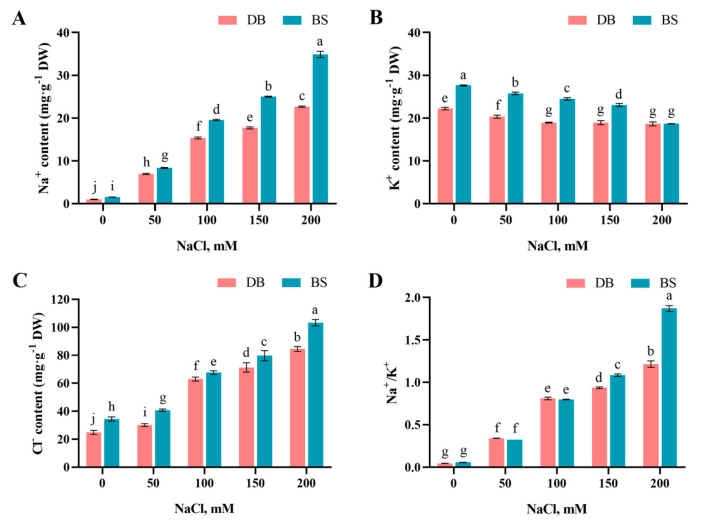
Effect of different NaCl concentrations on (**A**) Na^+^, (**B**) K^+^, (**C**) Cl^−^ and (**D**) Na^+^/K^+^ of *H. syriacus* ‘Duede Brabaul’ (DB) and *H. syriacus* ‘Blueberry Smoothie’ (BS). According to Duncan’s test, different lowercase letters (a–j) indicate significant differences (*p* < 0.05) between different treatments.

In general, with increasing NaCl concentration, the Na^+^ and Cl^−^ contents and Na^+^/K^+^ ratio in the leaves of the two hibiscus cultivars gradually increased, while the K^+^ content gradually decreased (Figure 3). Under different NaCl concentration treatments, the accumulation of Na^+^ and Cl^−^ in DB leaves was significantly lower than BS (*p* < 0.05), and Na^+^ content increases with increasing NaCl concentration, especially for BS. After NaCl treatment, the K^+^ content of DB decreased significantly (*p* < 0.05). When the NaCl concentration exceeded 100 mM, the K^+^ content in DB leaves remained unchanged, while the K^+^ content in BS leaves showed a downward trend with increasing NaCl concentration. The Na^+^/K^+^ ratio in the leaves of the two hibiscus cultivars increased significantly with increasing NaCl concentration (*p* < 0.05). Under 150 and 200 mM treatments, the Na^+^/K^+^ ratio in BS leaves was significantly higher than that of DB (*p* < 0.05). Under higher NaCl treatment, DB showed a smaller change in the Na^+^/K^+^ ratio and a stronger ability to maintain the balance of Na^+^ and K^+^ in the plants than BS.

### 2.5. Salt-Stress-Induced Reactive Oxygen Species Accumulation

**Figure 4:**
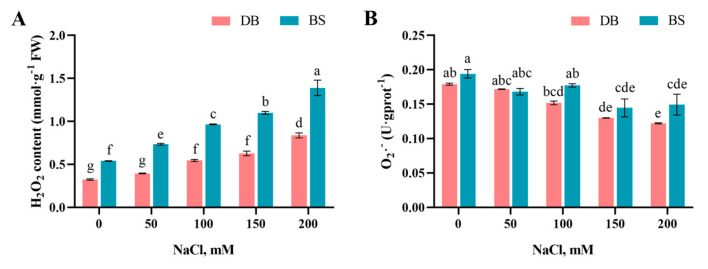
Effect of different NaCl concentrations on (**A**) hydrogen peroxide (H_2_O_2_) content, (**B**) inhibition of O_2_·^−^ product activity (O_2_·^−^) of *H. syriacus* ‘Duede Brabaul’ (DB) and *H. syriacus* ‘Blueberry Smoothie’ (BS). According to Duncan’s test, different lowercase letters (a–g) indicate significant differences (*p* < 0.05) between different treatments.

With increasing NaCl concentration, the H_2_O_2_ content in DB and BS leaves showed an overall upward trend (Figure 4A). Under both the control and NaCl treatments, the H_2_O_2_ content accumulation in BS leaves was significantly higher than that in DB leaves (*p* < 0.05). The inhibition of O_2_·^−^ product activity in both DB and BS leaves was significantly lower than that of the corresponding control plants only when the NaCl concentration was higher than 150 mM (Figure 4B), and there was no significant difference between DB and BS under equal NaCl concentration treatment (*p* > 0.05).

### 2.6. Salinity Modification of the Antioxidant System

**Figure 5:**
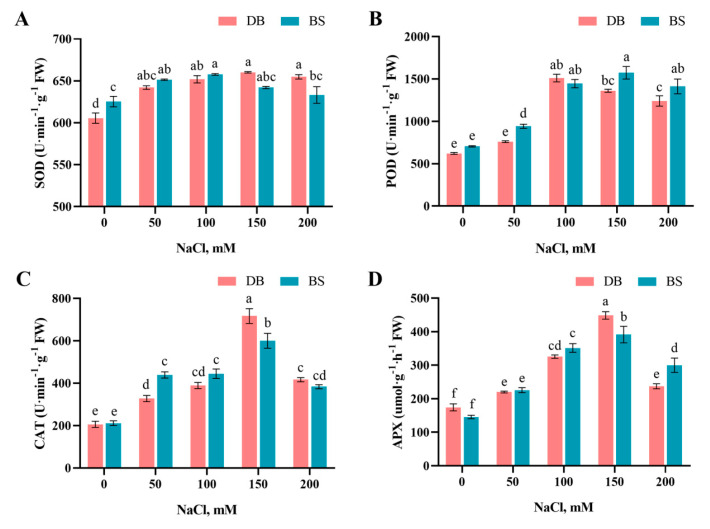
Effect of different NaCl concentrations on (**A**) superoxide dismutase activity (SOD), (**B**) peroxidase activity (POD), (**C**) catalase activity (CAT), and (**D**) ascorbate peroxidase activity (APX) of *H. syriacus* ‘Duede Brabaul’ (DB) and *H. syriacus* ‘Blueberry Smoothie’ (BS). According to Duncan’s test, different lowercase letters (a–f) indicate significant differences (*p* < 0.05) between different treatments.

To study the influence of salt stress on *Hibiscus syriacus*, a series of antioxidant enzymes responsible for ROS scavenging were quantified (Figure 5). The SOD, POD, CAT, and APX activities increased significantly in the two hibiscus cultivars after NaCl treatment. Under different NaCl concentrations, the SOD activity of the two hibiscus cultivars was significantly increased compared with CK (*p* < 0.05). With the exception that the SOD activity of DB leaves was significantly higher than that of BS at 200 mM, there was no significant difference between DB and BS under other NaCl concentration treatments. When the concentration of NaCl was 100 mM, the POD activity of DB and BS was significantly increased compared with that of CK (*p* < 0.05). With increasing NaCl concentration, the POD activity of BS did not change significantly, while the POD activity of DB showed a downward trend. The peaks of the CAT activity of DB and BS both appeared when the NaCl concentration was 150 mM, and they were increased by approximately 3.5 times and 2.8 times, respectively, compared with their respective CK. Under different NaCl concentrations, the changing trend of APX activity of DB and BS was basically similar to that of CAT, and both reached the maximum when the NaCl concentration was 150 mM, increased by 2.75 times and 2.68 times, respectively compared with their respective CK.

### 2.7. Salt Stress Induced Expression Level of AOX2 and ERD3

**Figure 6:**
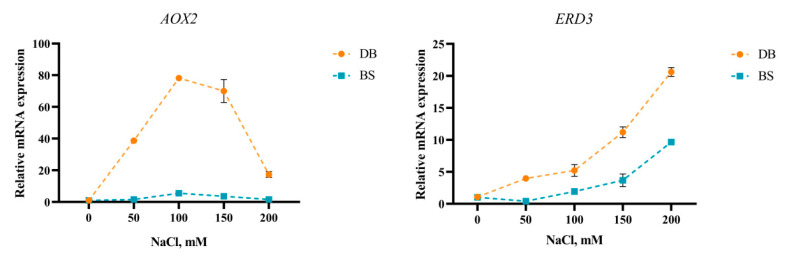
Effect of different NaCl concentrations on the expression levels of *AOX2* and *ERD3* of *H. syriacus* ‘Duede Brabaul’ (DB) and *H. syriacus* ‘Blueberry Smoothie’ (BS).

The expression levels of two key genes (*AOX2* and *ERD3*) associated with salt tolerance in leaves were investigated under different NaCl concentrations in the two cultivars of hibiscus, the results of which are shown in Figure 6. The lowest expression level was assumed as 1. The expression patterns of *AOX2* in DB and BS were basically the same, and the expression of *AOX2* was the highest at 100 mM treatment, and then decreased. The expression patterns of *ERD3* in DB and BS were similar. In contrast to *AOX2*, the expression of *EDR3* showed a significant upward trend with increasing NaCl concentration. The expression levels of genes involved in the salt-stress tolerance (*AOX2* and *ERD3*) were significantly higher in DB than that in BS under the different NaCl concentrations.

### 2.8. Comparison of Salt Tolerance in Two Hibiscus Cultivars

**Figure 7:**
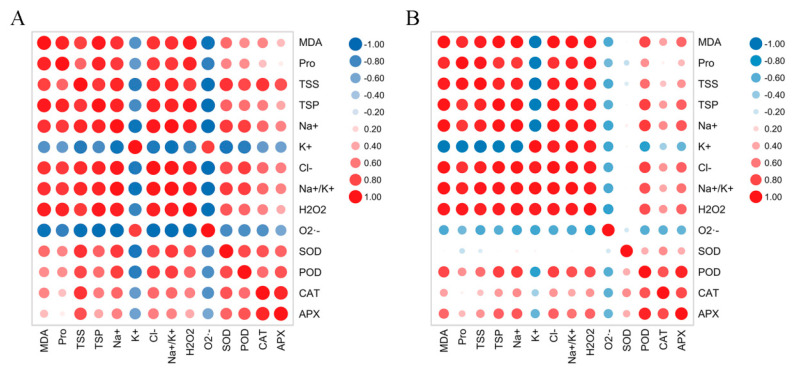
Correlation coefficients between physiological parameters, oxidative stress markers, and antioxidant capacity in (**A**) *H. syriacus* ‘Duede Brabaul’ (DB) and (**B**) *H. syriacus* ‘Blueberry Smoothie’ (BS) leaves. Plants were grown for 12 days at 5 salinity levels (0, 50, 100, 150, and 200 mM NaCl). Note: MDA, malondialdehyde; Pro, proline; TSS, total soluble sugars; TSP, total soluble protein; Na^+^, sodium ions; K^+^, potassium ions; Cl^−^, chloride ions; Na^+^/K^+^, sodium-to-potassium ion ratio; H_2_O_2_, hydrogen peroxide; O_2_·^−^, inhibition of O_2_·^−^ product activity; SOD, superoxide dismutase; POD, peroxidase; CAT, catalase; APX, ascorbate peroxidase.

In our study, we found a positive correlation between variables in general, with a few negative correlations. K^+^ and O_2_·^−^ showed a strong negative correlation with other variables in the two hibiscus cultivars. Under NaCl treatment, APX in the leaves of DB presented a weak positive correlation with MDA, Pro, TSP, and H_2_O_2_, and Pro presented a weak positive correlation with CAT, while APX in leaves of BS presented a weak positive correlation with Pro and SOD, and CAT also presented a weak positive correlation with some variables (Figure 7). In contrast to DB, SOD in BS leaves was not only very weakly correlated with MDA, TSP, Na^+^, and K^+^, but also weakly negatively correlated with other variables. Additionally, there was a strong positive correlation between the other variables of the two hibiscus cultivars under NaCl treatment.

**Figure 8:**
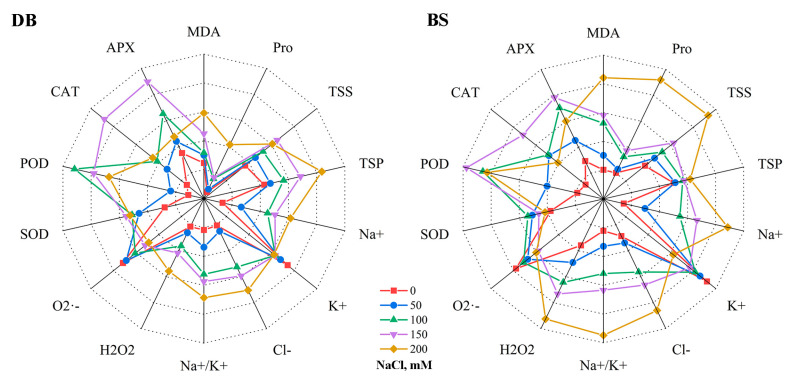
The radar chart of 14 indexes of *H. syriacus* ‘Duede Brabaul’ (DB) and *H. syriacus* ‘Blueberry Smoothie’ (BS) under NaCl stresses. Each point in this figure represents a specific measured value for each parameter without homogenization (see previous sections for detailed values). Due to the differences between the different parameters measured, the lengths of the different axis representations were adjusted to make the images more visible. The axis lengths and intersections are identical for the same parameters of the DB and BS. Note: MDA, malondialdehyde; Pro, proline; TSS, total soluble sugars; TSP, total soluble protein; Na^+^, sodium ions; K^+^, potassium ions; Cl^−^, chloride ions; Na^+^/K^+^, sodium-to- potassium ion ratio; H_2_O_2_, hydrogen peroxide; O_2_·^−^, inhibition of O_2_·^−^ product activity; SOD, superoxide dismutase; POD, peroxidase; CAT, catalase; APX, ascorbate peroxidase.

A comprehensive analysis of 14 indexes of the two hibiscus cultivars was made by radar charts (Figure 8). The overall trends of the indexes of DB and BS were similar except at 200 mM NaCl treatment. At 200 mM NaCl treatment, DB showed more stable performance in all indicators, and Na^+^/K^+^, and BS showed especially high Pro, TSP, and TSS accumulation when facing high Na^+^ toxicity. In general, under salt stress, plants maintained a normal internal working environment with the synergistic effect of ion uptake and the antioxidant system, which improved the tolerance. DB grew better under salt stress and had stronger salt tolerance than BS.

## 3. Discussion

The increases in Na^+^ content in hibiscus leaf tissues observed in this study confirmed the simulated salt stress gradient. The morphological characteristics of plants can directly reflect their growth status. Once a plant accumulates Na^+^ in the branches and is poisoned by Na^+^, the most obvious symptoms are wilting, yellowing, and shedding of leaves, and even death of the plant [30]. Plant salt tolerance is a complex biological phenomenon involving a variety of physiological and biochemical pathways [31]. It is of interest to study the tolerance and physiological mechanisms of two hibiscus cultivars in response to salt stress. In this study, chlorotic spots began to appear in BS plants when the NaCl concentration was 100 mM. These results showed that the phenotypic damage to the two hibiscus cultivar leaves was not obvious under NaCl treatment at a low concentration (50 mM). As the NaCl concentration increased, the leaves were chlorotic and curled, and even old leaves fell. However, DB can grow normally at 100 mM for 12 days. As the concentration of NaCl increases, the damage to DB is much less severe than that to the BS plants. This shows that the ability of DB plants to tolerate NaCl stress was higher than BS plants.

NaCl-induced ROS overproduction elevated lipid peroxidation, measured as MDA, which causes impaired membrane functions and properties [9]. In this study, the MDA content of the two hibiscus cultivars showed a significant increase with increasing NaCl concentration. This result is consistent with the effect of salt stress on honeysuckle [32] and potato [33]. The results of existing studies have shown that the degree of lipid peroxidation of salt-sensitive strains is higher than that of salt-tolerant strains [34]. Lower lipid peroxidation in DB seedlings relative to BS plants suggested that there may be an efficient antioxidant defense mechanism (see below) and/or favored membrane lipid remodeling [35] in DB, which most likely contributed to reduced lipid peroxidation and, in turn, maintenance of the cell membrane integrity under NaCl salinity.

After the osmotic balance in plant cells is broken, to maintain normal life activities, plants need to synthesize organic osmotic substances in the cell sap for osmotic adjustment to avoid or reduce damage [36]. Proline is a very important organic osmotic regulator that can balance the high concentration of salt in cells, prevent cell dehydration, and stabilize cell proteins, thereby preventing enzyme inactivation [37]. In this study, the proline content in the leaves of the two hibiscus cultivars gradually increased with increasing NaCl concentration, which is consistent with the results of eggplant [38] under NaCl stress. Under normal growth conditions, DB leaves with strong salt tolerance have less proline accumulation. Greater proline increased in the BS plants than in the DB plants under NaCl stress is most probably attributed to proline association with salt sensitivity reported in several plant species under saline conditions [39]. Soluble sugars have the effect of maintaining cell turgor [40]. The soluble sugar content in the leaves of hibiscus moscheutos significantly increased under both uniform and nonuniform salinity stress [41]. With increasing NaCl concentration, the soluble sugar content in rosemary leaves increased significantly [10]. In this study, when the NaCl concentration was lower than 150 mM, the soluble sugar content of DB and BS leaves showed a significant increase as the NaCl concentration increased. However, at a 200 mM NaCl concentration, the soluble sugar content of DB leaves with stronger salt tolerance decreased. Soluble sugars can be used as a carbon framework and energy source for other organic solutes [42]. It is speculated that DB leaves may initiate other, more effective, osmotic regulation mechanisms, such as soluble sugar as a substrate to synthesize other organic solutes. Soluble protein is an important osmotic adjustment substance in plants, and its content is an important reference value for plant adversity stress [43]. Under NaCl treatment, the soluble protein content of DB, which has stronger salt tolerance, increased more significantly than that of BS varieties, which was similar to that of lemon [44] and centipedegrass [45] under salt stress, indicating that DB can maintain the balance of osmotic regulation by increasing the content of soluble protein. Based on the distinct accumulation characteristics of these three kinds of osmotic adjustment substances under salt stress conditions, we speculate that the osmotic adjustment mechanisms of the two cultivars are different, although it needs to be studied in depth.

Except for osmotic stress, under high NaCl concentrations, the accumulation of noxious quantities of Na^+^ in plant cells disrupts normal physiological and biochemical activities, causing plants to suffer ion toxicity [40]. The Na^+^ content in the leaf cells of the two hibiscus cultivars increased with increasing NaCl concentration, which is similar to reports on Beta macrocarpa [36] and honeysuckle [32]. In this study, DB leaves accumulated less Na^+^ than BS leaves at the same NaCl concentration, which is consistent with the morphological characteristics of DB leaves that wilt less than BS leaves under NaCl stress. Under salt conditions, plants tend to accumulate more Na^+^ in the old leaves for survival [8]. Therefore, it is speculated that the old leaves of BS will fall off due to the accumulation of too much Na^+^ under high NaCl conditions. The excess accumulation of Na^+^ in plant cells will induce cytosolic K^+^ efflux and then lead to an imbalance in cellular homeostasis [46,47]. In this study, the K^+^ content in the leaves of the two hibiscus cultivars decreased with the increase in NaCl concentration, which is in contrast to that of intracellular Na^+^, implying that high NaCl concentration on the outside leads to an increase in Na^+^ content in cells, which in turn causes cytosolic K^+^ efflux in hibiscus leaves. This was consistent with results from sunflower [48] and castor [11]. Na^+^ competes with K^+^ in uptake and transport, which leads to K^+^ deficiency and Na^+^ excess, and the Na^+^/K^+^ ratio in the tissue increases significantly, thereby inhibiting plant growth [15]. K^+^ deficiency impacts the development of root components, such as the metaxylem, which is essential to the uptake of water and nutrients [49]. We found that with the increase in salt stress intensity, the change in K^+^ content in DB leaves was smaller than that in BS leaves, suggesting that high-affinity K^+^ uptake capacity might be present in DB plants under salt stress to give them stronger salt tolerance. Researchers have reached a consensus that the maintenance of a low cytosolic Na^+^/K^+^ ratio is critical for the adaptation of plants to salinity [46]. An increase in the Na^+^/K^+^ ratio was observed in both of the hibiscus cultivars, and an obviously higher Na^+^/K^+^ ratio was found in BS than in DB under higher salt concentrations, which demonstrated the relatively high salt tolerance in DB. Furthermore, different NaCl concentrations had a significant impact on the Cl^−^ content in hibiscus leaves, and the Cl^−^ content in DB and BS leaves increased with increasing NaCl concentration, which was similar to the patterns of atriplex [42] under NaCl stress. There was a strong correlation between Cl^−^ content and Na^+^ content in hibiscus leaves, and the increase of Cl^−^ may be necessary for electronic balance and maintaining a negative voltage under NaCl stress [50].

Under NaCl stress conditions, when the dynamic balance between ROS production and elimination necessary for normal cell homeostasis is broken, ROS can cause oxidative damage [51]. The main members of the ROS family include free radicals such as O_2_∙^−^ and OH∙ and nonradicals such as H_2_O_2_ and ^1^O_2_ [17,52]. Under the catalysis of SOD, O_2_∙^−^ will be disproportionated into reactive oxygen species (such as H_2_O_2_), generating highly reactive hydroxyl radicals (∙OH) [53,54]. O_2_∙^−^ and H_2_O_2_ destroy the redox balance in the plant, leading to cell membrane peroxidation and oxidative damage to the cell structure [55,56]. In this study, with increasing NaCl concentration, the H_2_O_2_ content of the two hibiscus leaves increased significantly, which is consistent with the results in japonica rice [57] under NaCl stress. The H_2_O_2_ content of salt-tolerant plants is lower than that of salt-sensitive plants under NaCl stress [58], which was also demonstrated in this study: compared with BS, the H_2_O_2_ content of DB leaves with stronger salt tolerance is lower under salt stress. Furthermore, the inhibition of O_2_·^−^ product activity of the two hibiscus cultivars was significantly lower than that of the control plants only under high NaCl concentration treatment. Moreover, the inhibition of O_2_·^−^ product activity between the two hibiscus cultivars was not significant, indicating that after 12 days of NaCl stress, most of the O_2_·^−^ in hibiscus leaves may have been converted to H_2_O_2_, which also aggravated the substantial increase in H_2_O_2_ content.

To address the adverse effects of excessive ROS, various antioxidative enzymes will be produced in plants [22]. Within the cell, SOD constitutes the first line of defense against ROS [56]. Under salt stress, the SOD activity of *Hibiscus moscheutos* [41] increased significantly compared with the control treatment. Similarly, higher SOD activities were measured in leaves of both hibiscus cultivars at higher salinity. We also found that the BS plants showed remarkably weaker SOD activity than the DB at the highest salinity level (200 mM). As SOD functions as a catalyst in the conversion of O_2_·^−^ to H_2_O_2_, we infer that there is less dismutation of O_2_·^−^ in high-salinity-stressed BS leaves than in DB leaves. POD can further remove H_2_O_2_, the antioxidant product of SOD in cells, and prevent more serious OH∙ toxicity [54]. Within a certain range of NaCl concentrations, NaCl can increase the POD activity in the leaves of lemon [44] and pistachio rootstock [59] and maintain the balance of ROS. The present study also showed a similar trend. Previous studies have shown that under NaCl stress, the APX activity of pistachio [60] leaves was strongly induced, and the activities of CAT and APX in the leaves of Momordica cochinchinensis [61] and *Glycine max* [62] were significantly higher than those of the control. In this study, with increasing NaCl concentration, the CAT and APX activities of the two hibiscus cultivars showed a single-peak curve, which was consistent with the results of tobacco under salt stress [63]. Both cultivars of hibiscus can reduce damage by increasing the activity of CAT and APX, except at the highest salinity level (200 mM). Our results suggest that POD, CAT, and APX synergistically regulate H_2_O_2_ detoxification under salt stress. Interestingly, the BS plants showed notably higher POD and APX activity than DB plants at the highest salinity level (200 mM), which is contrary to SOD activity. We infer that the extremely high accumulation of H_2_O_2_ at high salinity (200 mM) might result in stronger production of POD activity in BS than in DB. However, the cell membrane was still seriously damaged in high salinity-treated hibiscus. Hence, these ROS scavenging enzymes are still insufficient to scavenge the redundant ROS in high salt treatment.

Alternative oxidase (AOX) responds to various biological and abiotic stresses by maintaining the redox state and metabolic stability of plants [64]. AOX is activated at high respiratory substrate availability and is induced by high levels of endogenous or exogenous ROS [65]. In this study, the expression level of *AOX2* was significantly higher in DB leaves than in BS under salt stress. Similar findings have been reported by Sun et al. [66], which suggested that a high expression of *AOX2* induced an increased salt tolerance capability in Arabidopsis thaliana through the regulation of ROS. Under the osmotic pressure of long-term salt stress, plants are subjected to osmotic stress and to the following dehydration, which can induce the expression of a large number of early responses to dehydration (ERD) genes in plants [67]. The *ERD* genes could rapidly respond to dehydration and other abiotic stresses. Previous studies have demonstrated that *ERD3* was induced in *Hibiscus tiliaceus* in response to salt stress [68], which is similar to our study. In addition, the function of *ERD3* in salt tolerance has been confirmed in maize [69]. The expression level of *ERD3* in DB leaves was significantly higher than that in BS, which further confirmed that DB has stronger salt tolerance than BS at the transcriptional level.

## 4. Materials and Methods

### 4.1. Plant Materials and Growth Conditions

One-year cuttages *H. syriacus* ‘Duede Brabaul’ (DB) and *H. syriacus* ‘Blueberry Smoothie’ (BS), provided by Jiashan County Ecological Technology Company, China, were selected and used as experimental materials for this study. The experiment was executed in a greenhouse at the Experimental Station of Shandong Agricultural University (36°09′ N, 117°09′ E), Tai’an, China, at temperatures of 20 °C to 30 °C and humidity of approximately 60 ± 5%. The seedlings were exposed to natural sunlight. The seedlings were planted in plastic pots (6 cm × 5 cm × 8 cm) filled with growing media (peat soil/perlite/vermiculite = 1:1:1 *v/v*) until they were 15 cm tall. Then, they were moved to large plastic pots (20 cm × 20 cm × 18 cm). Each plant was planted in one pot and was periodically watered with tap water every 3 days.

### 4.2. Experimental Design and Salt Treatment

Before NaCl treatments were conducted, a preliminary experiment was performed in which *Hibiscus syriacus* L. plants (*H. syriacus* ‘Duede Brabaul’ (DB), *Hibiscus syriacus* f. paeoniflorus, *H. syriacus* ‘Blueberry Smoothie’ (BS), *H. syriacus* ‘Wood Bridge’, and *H. syriacus* ‘Lavender Chiffon’) were treated with five NaCl concentrations for 15 days to evaluate their overall salinity tolerance. Seedlings of two hibiscus cultivars (DB and BS) with distinct salt tolerance were selected for the formal salt stress experiment according to the results of the preliminary experiment. To accurately control the salt concentration, the solution culture method was adopted.

Hibiscus plants with a height of 25 cm and approximately 10 leaves growing healthily and with the same growth trend were selected, removed from their soil plastic pot, washed with tap water, fixed on Kt boards with cotton, and placed in plastic pots (38 cm × 29 cm × 12 cm), transplanted with 6 trees in each pot, and cultured with water for 1 day. Then, the seedlings were cultured in 1/2 Hoagland nutrient solution [70] for 3 weeks. After the seedlings returned to normal growth, robust and consistent seedlings were selected and transferred to nutrient solution containing 0, 50, 100, 150, and 200 mM NaCl (the solution was changed every three days) with root salt stress treatment for 12 days. Four plants were used for each treatment. Each treatment had three biological replicates. The samples of fresh leaves were stored at −80 °C.

### 4.3. Determination of Physiological and Biochemical Indicators

#### 4.3.1. MDA Content Determination

Lipid peroxidation was measured by determining the level of malondialdehyde (MDA) using the technique of Kato and Shimizu [63]. First, 5 mL 5% (*w/v*) trichloroacetic acid (TCA) was added into 0.5 g fresh leaves, and the homogenate was centrifuged at 3000 r·min^−1^ for 10 min. Then, 2 mL 0.67% (*w/v*) thiobarbituric acid (TBA) was added to 2 mL supernatant, boiled in a 100 °C water bath for 30 min, and then centrifuged once after cooling. Finally, the absorbance was measured at 450 nm, 532 nm, and 600 nm, respectively.

#### 4.3.2. Proline Content, Soluble Sugar, and Soluble Protein Determination

Dry leaf samples were used for proline extraction, homogenized in 3% (*w/v*) sulfosalicylic acid. The proline content was determined according to Abdallah et al. [71] using L-proline for the standard curve. Moreover, the changes in proline contents were recorded on a 722N-spectrophotometer (Shanghai Youke, Shanghai, China) at 520 nm. Additionally, the proline contents were expressed in μg·g^−1^ of fresh leaf weight.

The content of soluble sugars in the leaves was determined according to the method of Kumar et al. [72]. A quantity of 0.2 g fresh leaves was added to 10 mL distilled water and bathed in boiling water for 30 min. The extract was filtered into a 50 mL volumetric flask, and the volume was constant to the scale line. A quantity of 1 mL extract was added to 1 mL distilled water, and then 0.5 mL ethyl anthrone acetate and 5 mL concentrated sulfuric acid were added in sequence. After the full shock, the extract was immediately placed in a boiling water bath for 1 min and cooled at 620 nm.

For the analysis of soluble protein, 0.1 g of fresh leaves was ground evenly on ice with 1 mL phosphate buffer (PBS, pH 7.8). The homogenate was centrifuged at 12,000 r·min^−1^ at 4 °C for 10 min, 0.1 mL supernatant was absorbed, and 5 mL Coomachus bright blue G-250 was added. The absorbance was measured at 595 nm and bovine serum protein (BSA) was used as the standard protein [73].

#### 4.3.3. H_2_O_2_ Content and Inhibition of O_2_·^−^ Product Activity Determination

H_2_O_2_ content was determined according to the kit of Nanjing Jiancheng Institute of Biological Engineering (www.njjcbio.com No. A064-1-1), and inhibition of O_2_·^−^ product activity was determined according to the kit of Nanjing Jiancheng Institute of Biological Engineering (www.njjcbio.com No. A052-1-1) in strict accordance with the instructions for operation.

#### 4.3.4. Antioxidant Enzyme Activity Determination

Superoxide dismutase activity (SOD, EC.1.15.1.1) was quantified using the NBT assay according to the method described by Ibrahim et al. [74]. Peroxidase activity (POD, EC: 1.11.1.7) was estimated as outlined in Wu et al. [75], and the reaction solution contained 0.4 mL of guaiacol (20 mm), 2 mL of 7.8 pH sodium phosphate buffer (50 mm), 0.5 mL of hydrogen peroxide (40 mm), and 0.1 mL of enzyme extract. The absorbances for catalase and peroxidase were measured at wavelengths of 240 nm and 470 nm, respectively. Catalase activity (CAT, EC 1.11.1.6) was monitored by tracking H_2_O_2_ consumption at 240 nm for 2 min [63]. Ascorbate peroxidase activity (APX, EC 1.11.1.11) was measured by following the protocol of Chen et al. [76]. The changes in the APX assay were computed by spectrophotometry at 290 nm for 100 s.

### 4.4. Determination of Ion Contents

The contents (mg·g^−1^ dry weight; DW) of sodium (Na^+^) and potassium (K^+^) in dried leaves of hibiscus were determined according to Voigt et al. [77]. Finally, the contents were measured by flame spectrophotometry (FP6400, Shanghai Yuefeng, Shanghai, China). Furthermore, the Na^+^/K^+^ ratio was obtained after dividing the potassium ion content by the sodium ion content. The chloride ion (Cl^−^) concentration was determined through titration with AgNO_3_ according to the method of Jamshidi et al. [59].

### 4.5. RNA Extraction and Quantitative PCR Analysis

Total RNA was isolated with a Trizol reagent (TransGen Biotech, Beijing, China). The RNA preparation was then treated with DNase I. First-strand cDNA was synthesized from 2 μg of total RNA using the PrimeScript™ RT reagent Kit with gDNA Eraser (TransGen Biotech, Beijing, China). The hibiscus 18S rRNA gene was used as the reference gene. Quantitative real-time PCR (qRT-PCR) and data analyses were performed as described previously [78]. All primers used are listed in Supplementary Table S1.

### 4.6. Data Analysis

The data are represented as the means ± standard errors (M ± SE). We subjected all data to a one-way analysis of variance (ANOVA), and significant differences among the means were tested by Duncan’s multiple range test (*p* < 0.05) via SPSS 26.0. The interrelationships among the data were examined by Pearson’s correlation coefficient (*r*). Charts were plotted using GraphPad Prism 8.0 software.

## 5. Conclusions

This research reveals that salt stress affects two hibiscus cultivars differently, and they adapt to the stress through different regulations. The salt-tolerant cultivar DB achieves its high tolerance by limiting Na^+^ uptake and ensuring K^+^ homeostasis, avoiding the toxicity of Na^+^ under salt stress, and meeting the normal nutrient requirements. The activities of the antioxidant enzymes SOD, POD, CAT, and APX in hibiscus leaves increased under Na^+^ stress, thus improving the scavenging of harmful substances such as ROS (H_2_O_2_, O_2_·^−^) and MDA. Meanwhile, *AOX2* and *ERD3* in DB leaves were significantly induced under Na^+^ stress, which was consistent with their stable antioxidant and osmotic regulation (Pro, TSS, TSP) system. Overall, the cooperation of multiple systems improves the tolerance of hibiscus plants to salt stress and ensures normal physiological metabolism.

## Data Availability

All data relevant to the main findings of this study are included within the article.

## Supplementary Materials

The following supporting information can be downloaded at: https://www.mdpi.com/article/10.3390/plants12071525/s1, Table S1: Primer sequences for quantitative real-time PCR.

## References

[1] Parida A.K., Das A.B. (2005). Salt Tolerance and Salinity Effects on Plants: A Review. Ecotoxicol. Environ. Saf..

[2] Deinlein U., Stephan A.B., Horie T., Luo W., Xu G., Schroeder J.I. (2014). Plant Salt-Tolerance Mechanisms. Trends Plant Sci..

[3] Munns R., Tester M. (2008). Mechanisms of Salinity Tolerance. Annu. Rev. Plant Biol..

[4] Zhao G., Shi Q., Han Y., Li S., Wang W. (2014). The Physiological and Biochemical Responses of a Medicinal Plant (*Salvia miltiorrhiza* L.) to Stress Caused by Various Concentrations of NaCl. PLoS ONE.

[5] Chen J., Zhang J., Hu J., Xiong W., Du C., Lu M. (2017). Integrated Regulatory Network Reveals the Early Salt Tolerance Mechanism of *Populus euphratica*. Sci. Rep..

[6] Yao X., Zhou M., Ruan J., Peng Y., Ma C., Wu W., Gao A., Weng W., Cheng J. (2022). Physiological and Biochemical Regulation Mechanism of Exogenous Hydrogen Peroxide in Alleviating NaCl Stress Toxicity in Tartary Buckwheat (*Fagopyrum tataricum* (L.) Gaertn). Int. J. Mol. Sci..

[7] Patel J., Khandwal D., Choudhary B., Ardeshana D., Jha R.K., Tanna B., Yadav S., Mishra A., Varshney R.K., Siddique K.H.M. (2022). Differential Physio-Biochemical and Metabolic Responses of Peanut (*Arachis hypogaea* L.) under Multiple Abiotic Stress Conditions. Int. J. Mol. Sci..

[8] Munns R., Gilliham M. (2015). Salinity Tolerance of Crops—What Is the Cost?. New Phytol..

[9] Chen Y.-E., Mao J.-J., Sun L.-Q., Huang B., Ding C.-B., Gu Y., Liao J.-Q., Hu C., Zhang Z.-W., Yuan S. (2018). Exogenous Melatonin Enhances Salt Stress Tolerance in Maize Seedlings by Improving Antioxidant and Photosynthetic Capacity. Physiol. Plant..

[10] Chetouani M., Mzabri I., Aamar A., Boukroute A., Kouddane N., Berrichi A. (2019). Morphological-Physiological and Biochemical Responses of Rosemary (*Rosmarinus officinalis*) to Salt Stress. Mater. Today Proc..

[11] Rodrigues C.R.F., Silva E.N., da Mata Moura R., dos Anjos D.C., Hernandez F.F.F., Viégas R.A. (2014). Physiological Adjustment to Salt Stress in R. Communis Seedlings Is Associated with a Probable Mechanism of Osmotic Adjustment and a Reduction in Water Lost by Transpiration. Ind. Crops Prod..

[12] Qu C., Liu C., Gong X., Li C., Hong M., Wang L., Hong F. (2012). Impairment of Maize Seedling Photosynthesis Caused by a Combination of Potassium Deficiency and Salt Stress. Environ. Exp. Bot..

[13] Flowers T.J., Colmer T.D. (2008). Salinity Tolerance in Halophytes. New Phytol..

[14] Chakraborty K., Bose J., Shabala L., Eyles A., Shabala S. (2016). Evaluating Relative Contribution of Osmotolerance and Tissue Tolerance Mechanisms toward Salinity Stress Tolerance in Three Brassica Species. Physiol. Plant..

[15] Basu S., Kumar A., Benazir I., Kumar G. (2021). Reassessing the Role of Ion Homeostasis for Improving Salinity Tolerance in Crop Plants. Physiol. Plant..

[16] Das K., Roychoudhury A. (2014). Reactive Oxygen Species (ROS) and Response of Antioxidants as ROS-Scavengers during Environmental Stress in Plants. Front. Environ. Sci..

[17] Mittler R. (2002). Oxidative Stress, Antioxidants and Stress Tolerance. Trends Plant Sci..

[18] Tarchoune I., Sgherri C., Izzo R., Lachaâl M., Navari-Izzo F., Ouerghi Z. (2012). Changes in the Antioxidative Systems of *Ocimum basilicum* L. (Cv. Fine) under Different Sodium Salts. Acta Physiol. Plant.

[19] Huihui Z., Xin L., Yupeng G., Mabo L., Yue W., Meijun A., Yuehui Z., Guanjun L., Nan X., Guangyu S. (2020). Physiological and Proteomic Responses of Reactive Oxygen Species Metabolism and Antioxidant Machinery in Mulberry (*Morus alba* L.) Seedling Leaves to NaCl and NaHCO_3_ Stress. Ecotoxicol. Environ. Saf..

[20] Meloni D.A., Oliva M.A., Martinez C.A., Cambraia J. (2003). Photosynthesis and Activity of Superoxide Dismutase, Peroxidase and Glutathione Reductase in Cotton under Salt Stress. Environ. Exp. Bot..

[21] Moradi F., Ismail A.M. (2007). Responses of Photosynthesis, Chlorophyll Fluorescence and ROS-Scavenging Systems to Salt Stress During Seedling and Reproductive Stages in Rice. Ann. Bot..

[22] Ashraf M., Harris P.J.C. (2004). Potential Biochemical Indicators of Salinity Tolerance in Plants. Plant Sci..

[23] Sarker U., Oba S. (2020). The Response of Salinity Stress-Induced A. Tricolor to Growth, Anatomy, Physiology, Non-Enzymatic and Enzymatic Antioxidants. Front. Plant Sci..

[24] Eo H.J., Kwon H.Y., Da Kim S., Kang Y., Park Y., Park G.H. (2020). GC/MS Analysis and Anti-Inflammatory Effect of Leaf Extracts from *Hibiscus syriacus* through Inhibition of NF-ΚB and MAPKs Signaling in LPS-Stimulated RAW264.7 Macrophages. Plant Biotechnol. Rep..

[25] Hsu R.-J., Hsu Y.-C., Chen S.-P., Fu C.-L., Yu J.-C., Chang F.-W., Chen Y.-H., Liu J.-M., Ho J.-Y., Yu C.-P. (2015). The Triterpenoids of *Hibiscus syriacus* Induce Apoptosis and Inhibit Cell Migration in Breast Cancer Cells. BMC Complement. Altern. Med..

[26] Karunarathne W.A.H.M., Lee K.T., Choi Y.H., Jin C.-Y., Kim G.-Y. (2020). Anthocyanins Isolated from *Hibiscus syriacus* L. Attenuate Lipopolysaccharide-Induced Inflammation and Endotoxic Shock by Inhibiting the TLR4/MD2-Mediated NF-ΚB Signaling Pathway. Phytomedicine.

[27] Kim Y.H., Im A.-R., Park B.-K., Paek S.H., Choi G., Kim Y.R., Whang W.K., Lee K.H., Oh S.-E., Lee M.Y. (2018). Antidepressant-Like and Neuroprotective Effects of Ethanol Extract from the Root Bark of *Hibiscus syriacus* L.. BioMed Res. Int..

[28] Teets T.M., Hummel R.L., Guy C.L. (1989). Cold-Acclimation of *Hibiscus rosa-sinensis* L. and *Hibiscus syriacus* L. in Natural and Controlled Environments. Plant Cell Environ..

[29] Gadwal R., Naik G.R. (2014). A Comparative Study on the Effect of Salt Stress on Seed Germination and Early Seedling Growth of Two Hibiscus Species. IOSR-JAVS.

[30] Negrão S., Schmöckel S.M., Tester M. (2017). Evaluating Physiological Responses of Plants to Salinity Stress. Ann. Bot..

[31] Khan M.A.H., Mia A.B., Quddus A., Sarker K.K., Rahman M., Skalicky M., Brestic M., Gaber A., Alsuhaibani A.M., Hossain A. (2022). Salinity-Induced Physiological Changes in Pea (*Pisum sativum* L.): Germination Rate, Biomass Accumulation, Relative Water Content, Seedling Vigor and Salt Tolerance Index. Plants.

[32] He W., Yan K., Zhang Y., Bian L., Mei H., Han G. (2021). Contrasting Photosynthesis, Photoinhibition and Oxidative Damage in Honeysuckle (*Lonicera japonica* Thunb.) under Iso-Osmotic Salt and Drought Stresses. Environ. Exp. Bot..

[33] Li Q., Qin Y., Hu X., Jin L., Li G., Gong Z., Xiong X., Wang W. (2022). Physiology and Gene Expression Analysis of Potato (*Solanum tuberosum* L.) in Salt Stress. Plants.

[34] Juan M., Rivero R., Romero L., Ruiz J. (2005). Evaluation of Some Nutritional and Biochemical Indicators in Selecting Salt-Resistant Tomato Cultivars. Environ. Exp. Bot..

[35] Mansour M.M.F., Salama K.H.A., Allam H.Y.H. (2015). Role of the Plasma Membrane in Saline Conditions: Lipids and Proteins. Bot. Rev..

[36] Hamouda I., Badri M., Mejri M., Cruz C., Siddique K.H.M., Hessini K. (2016). Salt Tolerance of Beta Macrocarpa Is Associated with Efficient Osmotic Adjustment and Increased Apoplastic Water Content. Plant Biol..

[37] Jafari S., Hashemi Garmdareh S.E. (2019). Effects of Salinity on Morpho-Physiological, and Biochemical Characteristics of Stock Plant (*Matthiola incana* L.). Sci. Hortic..

[38] Hannachi S., Van Labeke M.-C. (2018). Salt Stress Affects Germination, Seedling Growth and Physiological Responses Differentially in Eggplant Cultivars (*Solanum melongena* L.). Sci. Hortic..

[39] Mansour M.M.F., Ali E.F. (2017). Evaluation of Proline Functions in Saline Conditions. Phytochemistry.

[40] Abid M., Zhang Y.J., Li Z., Bai D.F., Zhong Y.P., Fang J.B. (2020). Effect of Salt Stress on Growth, Physiological and Biochemical Characters of Four Kiwifruit Genotypes. Sci. Hortic..

[41] Feng X., Hussain T., Guo K., An P., Liu X. (2021). Physiological, Morphological and Anatomical Responses of *Hibiscus moscheutos* to Non-Uniform Salinity Stress. Environ. Exp. Bot..

[42] Hussin S., Geissler N., Koyro H.-W. (2013). Effect of NaCl Salinity on *Atriplex nummularia* (L.) with Special Emphasis on Carbon and Nitrogen Metabolism. Acta Physiol. Plant..

[43] Luis Castañares J., Alberto Bouzo C. (2019). Effect of Exogenous Melatonin on Seed Germination and Seedling Growth in Melon (*Cucumis melo* L.) Under Salt Stress. Hortic. Plant J..

[44] Khalid M.F., Hussain S., Anjum M.A., Ahmad S., Ali M.A., Ejaz S., Morillon R. (2020). Better Salinity Tolerance in Tetraploid vs Diploid Volkamer Lemon Seedlings Is Associated with Robust Antioxidant and Osmotic Adjustment Mechanisms. J. Plant Physiol..

[45] Li J., Ma J., Guo H., Zong J., Chen J., Wang Y., Li D., Li L., Wang J., Liu J. (2018). Growth and Physiological Responses of Two Phenotypically Distinct Accessions of Centipedegrass (*Eremochloa ophiuroides* (Munro) Hack.) to Salt Stress. Plant Physiol. Biochem..

[46] Assaha D.V.M., Ueda A., Saneoka H., Al-Yahyai R., Yaish M.W. (2017). The Role of Na^+^ and K^+^ Transporters in Salt Stress Adaptation in Glycophytes. Front. Physiol..

[47] Zhang T., Sun K., Chang X., Ouyang Z., Meng G., Han Y., Shen S., Yao Q., Piao F., Wang Y. (2022). Comparative Physiological and Transcriptomic Analyses of Two Contrasting Pepper Genotypes under Salt Stress Reveal Complex Salt Tolerance Mechanisms in Seedlings. Int. J. Mol. Sci..

[48] Gogna M., Choudhary A., Mishra G., Kapoor R., Bhatla S.C. (2020). Changes in Lipid Composition in Response to Salt Stress and Its Possible Interaction with Intracellular Na^+^-K^+^ Ratio in Sunflower (*Helianthus annuus* L.). Environ. Exp. Bot..

[49] Silva B.R.S., Batista B.L., Lobato A.K.S. (2021). Anatomical Changes in Stem and Root of Soybean Plants Submitted to Salt Stress. Plant Biol..

[50] Teakle N.L., Tyerman S.D. (2010). Mechanisms of Cl^−^ Transport Contributing to Salt Tolerance. Plant Cell Environ..

[51] Liu Y., Ji D., Turgeon R., Chen J., Lin T., Huang J., Luo J., Zhu Y., Zhang C., Lv Z. (2019). Physiological and Proteomic Responses of Mulberry Trees (*Morus alba* L.) to Combined Salt and Drought Stress. Int. J. Mol. Sci..

[52] de Oliveira V.P., Lima M.D.R., da Silva B.R.S., Batista B.L., da Silva Lobato A.K. (2019). Brassinosteroids Confer Tolerance to Salt Stress in *Eucalyptus urophylla* Plants Enhancing Homeostasis, Antioxidant Metabolism and Leaf Anatomy. J. Plant Growth Regul..

[53] Nie W., Gong B., Chen Y., Wang J., Wei M., Shi Q. (2018). Photosynthetic Capacity, Ion Homeostasis and Reactive Oxygen Metabolism Were Involved in Exogenous Salicylic Acid Increasing Cucumber Seedlings Tolerance to Alkaline Stress. Sci. Hortic..

[54] Sarabi B., Bolandnazar S., Ghaderi N., Ghashghaie J. (2017). Genotypic Differences in Physiological and Biochemical Responses to Salinity Stress in Melon (*Cucumis melo* L.) Plants: Prospects for Selection of Salt Tolerant Landraces. Plant Physiol. Biochem..

[55] Foyer C.H. (1996). Oxygen Processing in Photosynthesis. Biochem. Soc. Trans..

[56] Gill S.S., Tuteja N. (2010). Reactive Oxygen Species and Antioxidant Machinery in Abiotic Stress Tolerance in Crop Plants. Plant Physiol. Biochem..

[57] Ye S., Huang Z., Zhao G., Zhai R., Ye J., Wu M., Yu F., Zhu G., Zhang X. (2021). Differential Physiological Responses to Salt Stress between Salt-Sensitive and Salt-Tolerant *japonica* Rice Cultivars at the Post-Germination and Seedling Stages. Plants.

[58] Hichem H., Mounir D., Naceur E.A. (2009). Differential Responses of Two Maize (*Zea mays* L.) Varieties to Salt Stress: Changes on Polyphenols Composition of Foliage and Oxidative Damages. Ind. Crops Prod..

[59] Jamshidi Goharrizi K., Amirmahani F., Salehi F. (2020). Assessment of Changes in Physiological and Biochemical Traits in Four Pistachio Rootstocks under Drought, Salinity and Drought + Salinity Stresses. Physiol. Plant..

[60] Bagheri M., Gholami M., Baninasab B. (2019). Hydrogen Peroxide-Induced Salt Tolerance in Relation to Antioxidant Systems in Pistachio Seedlings. Sci. Hortic..

[61] Jumpa T., Beckles D.M., Songsri P., Pattanagul K., Pattanagul W. (2022). Physiological and Metabolic Responses of Gac Leaf (*Momordica cochinchinensis* (Lour.) Spreng.) to Salinity Stress. Plants.

[62] Da Silva B.R.S., Lobato E.M.S.G., dos Santos L.A., Pereira R.M., Batista B.L., Alyemeni M.N., Ahmad P., Lobato A.K.D.S. (2023). How Different Na+ Concentrations Affect Anatomical, Nutritional Physiological, Biochemical, and Morphological Aspects in Soybean Plants: A Multidisciplinary and Comparative Approach. Agronomy.

[63] Kato M., Shimizu S. (1987). Chlorophyll Metabolism in Higher Plants. VII. Chlorophyll Degradation in Senescing Tobacco Leaves; Phenolic-Dependent Peroxidative Degradation. Can. J. Bot..

[64] Analin B., Mohanan A., Bakka K., Challabathula D. (2020). Cytochrome Oxidase and Alternative Oxidase Pathways of Mitochondrial Electron Transport Chain Are Important for the Photosynthetic Performance of Pea Plants under Salinity Stress Conditions. Plant Physiol. Biochem..

[65] Polidoros A.N., Mylona P.V., Arnholdt-Schmitt B. (2009). Aox Gene Structure, Transcript Variation and Expression in Plants. Physiol. Plant..

[66] Sun L.J., Zhao X.Y., Ren J., Yan S.P., Zhao X.Y., Song X.S. (2021). Overexpression of *Cerasus humilis* ChAOX2 Improves the Tolerance of Arabidopsis to Salt Stress. 3 Biotech.

[67] Alves M.S., Reis P.A.B., Dadalto S.P., Faria J.A.Q.A., Fontes E.P.B., Fietto L.G. (2011). A Novel Transcription Factor, ERD15 (Early Responsive to Dehydration 15), Connects Endoplasmic Reticulum Stress with an Osmotic Stress-Induced Cell Death Signal. J. Biol. Chem..

[68] Yang G., Zhou R., Tang T., Chen X., Ouyang J., He L., Li W., Chen S., Guo M., Li X. (2011). Gene Expression Profiles in Response to Salt Stress in *Hibiscus tiliaceus*. Plant Mol. Biol. Rep..

[69] Song X., Weng Q., Zhao Y., Ma H., Song J., Su L., Yuan J., Liu Y. (2018). Cloning and Expression Analysis of ZmERD3 Gene From Zea Mays. Iran. J. Biotechnol..

[70] Hoagland D.R., Arnon D.I. (1950). The Water-Culture Method for Growing Plants without Soil.

[71] Ben Abdallah M., Trupiano D., Polzella A., De Zio E., Sassi M., Scaloni A., Zarrouk M., Ben Youssef N., Scippa G.S. (2018). Unraveling Physiological, Biochemical and Molecular Mechanisms Involved in Olive (*Olea europaea* L. Cv. Chétoui) Tolerance to Drought and Salt Stresses. J. Plant Physiol..

[72] Kumar S., Li G., Huang X., Ji Q., Zhou K., Hou H., Ke W., Yang J. (2021). Phenotypic, Nutritional, and Antioxidant Characterization of Blanched *Oenanthe javanica* for Preferable Cultivar. Front. Plant Sci..

[73] Bradford M.M. (1976). A Rapid and Sensitive Method for the Quantitation of Microgram Quantities of Protein Utilizing the Principle of Protein-Dye Binding. Anal. Biochem..

[74] Ibrahim W., Qiu C.-W., Zhang C., Cao F., Shuijin Z., Wu F. (2019). Comparative Physiological Analysis in the Tolerance to Salinity and Drought Individual and Combination in Two Cotton Genotypes with Contrasting Salt Tolerance. Physiol. Plant..

[75] Wu F., Zhang G., Dominy P. (2003). Four Barley Genotypes Respond Differently to Cadmium: Lipid Peroxidation and Activities of Antioxidant Capacity. Environ. Exp. Bot..

[76] Chen F., Wang F., Wu F., Mao W., Zhang G., Zhou M. (2010). Modulation of Exogenous Glutathione in Antioxidant Defense System against Cd Stress in the Two Barley Genotypes Differing in Cd Tolerance. Plant Physiol. Biochem..

[77] Voigt E.L., Caitano R.F., Maia J.M., Ferreira-Silva S.L., De Macêdo C.E.C., Silveira J.A.G. (2009). Involvement of Cation Channels and NH_4_^+^-Sensitive K^+^ Transporters in Na^+^ Uptake by Cowpea Roots under Salinity. Biol. Plant..

[78] Wang X., Li J., Guo J., Qiao Q., Guo X., Ma Y. (2020). The WRKY transcription factor PlWRKY65 enhances the resistance of *Paeonia lactiflora* (herbaceous peony) to *Alternaria tenuissima*. Hortic. Res..

