# Supplementary material for: Comparative Analysis of Antioxidant System and Salt-Stress Tolerance in Two Hibiscus Cultivars Exposed to NaCl Toxicity
Source: Plants (Basel). 2023 Mar 31;12(7):1525. doi: 10.3390/plants12071525 (PMC10097027; doi:10.3390/plants12071525)
Supplement: Supplementary file 1 [file plants-12-01525-s001.zip › plants-2309533-supplementary.pdf]

Supplementary Table S1 Primer sequences for quantitative real-time PCR.

Gene ID	Gene name	Primer sequence (5'-3')
KM117267.1	18s RNA	F:AACACGGACCAAGGAGTCTG
		R:GCCTCCACCAGAGTTTCCTC
LOC120201429	AOX2	F:ATGACGTTCATGGAGGTGTCC
		R:TCCTCAAGGTATCCCACCACT
HO762719	ERD3	F: TGTTATCGACGATCCCCTATGG
		R: TGGCTCTCGGCAGTGAAGA
